# Quantitative assessment of geniohyoid before and after rehabilitation in stroke patients with dysphagia by ultrasound

**DOI:** 10.3389/fresc.2026.1776612

**Published:** 2026-06-17

**Authors:** Jun Liu, Lin Gu, Li Huang, Yanyan Lin, Yunyun Hu

**Affiliations:** 1 Department of Ultrasound, Ruijin Hospital, Shanghai Jiao Tong University School of Medicine, Shanghai, China; 2 Department of Ultrasound, Shanghai Ruijin Rehabilitation Hospital, Shanghai, China; 3 Department of Rehabilitation Medicine, Shanghai Ruijin Rehabilitation Hospital, Shanghai, China

**Keywords:** dysphagia, geniohyoid, rehabilitation, stroke, ultrasound

## Abstract

**Objective:**

To quantitatively assess geniohyoid movement patterns using ultrasonography in stroke patients with dysphagia before and after rehabilitation.

**Methods:**

This study included 30 post-stroke dysphagia patients undergoing rehabilitation and 30 healthy controls. Ultrasonographic parameters of geniohyoid movement during swallowing were compared: (1) between patients (pre-treatment) and controls, (2) within patients pre- vs. post-treatment, and (3) between patients (post-treatment) and controls.

**Results:**

Pretreatment, significant differences were observed between patients and controls in geniohyoid displacement (*P* < 0.05) as well as in mean movement velocity and duration (*P* < 0.01). Post-rehabilitation, patients showed significant improvements in all three parameters compared to baseline (*P* < 0.05). While post-treatment displacement normalized to control levels (*P* > 0.05), mean velocity and movement duration remained significantly different from controls (*P* < 0.05).

**Conclusion:**

Ultrasonography provides an effective quantitative method for evaluating geniohyoid motility in stroke-related dysphagia, demonstrating measurable rehabilitation-induced changes in movement dynamics.

## Introduction

Post-stroke dysphagia is highly prevalent, affecting 27%–64% of patients (1). This condition not only elevates mortality risk but also significantly impairs quality of life (2). Currently, dysphagia screening and assessment rely on three primary approaches: swallowing function scales, clinical swallowing evaluations, and instrumental examinations (3). Among instrumental methods, videofluoroscopic swallowing study (VFSS) and fiberoptic endoscopic evaluation of swallowing (FEES) are the most widely utilized in clinical practice. Although VFSS is regarded as the gold standard for dysphagia diagnosis, it has notable limitations, including radiation exposure, poor short-term repeatability, and potential postoperative complications. These constraints make VFSS less feasible for elderly stroke patients or those with severe conditions (4).

Similarly, FEES allows direct visualization of pharyngeal and laryngeal structures during swallowing, providing valuable information about bolus passage, airway protection, and secretion management. However, FEES is invasive, cannot visualize the oral or esophageal phase, and has a brief “white-out” period at the moment of swallow, which may obscure key events. Despite these limitations, FEES is often preferred for bedside assessment and for patients who cannot be transported to radiology suites (4).

To better understand the role of the geniohyoid muscle in swallowing, a brief overview of the swallowing mechanism is helpful. Normal deglutition involves four coordinated phases: oral preparatory, oral propulsive, pharyngeal, and esophageal. During the pharyngeal phase, the hyoid bone and larynx move upward and anteriorly, which helps close the airway and open the upper esophageal sphincter (UES). The geniohyoid muscle, a key suprahyoid muscle, contributes not only to anterior hyoid displacement and UES opening but also to posterior propulsion of the bolus by elevating and moving the hyoid forward, thereby facilitating epiglottic inversion and laryngeal vestibule closure (5). Additionally, its contraction assists in tilting the thyroid cartilage and stretching the pharyngeal wall, which aids in clearing the bolus from the hypopharynx.

In contrast, ultrasonography offers real-time imaging with high sensitivity for dynamic observation. Previous studies have demonstrated the critical role of the geniohyoid muscle in hyoid displacement, and its superficial location allows for clear ultrasonographic visualization (6). Recently, ultrasound has gained increasing attention as a non-invasive, radiation-free tool for assessing swallowing muscle function in stroke patients (7, 8). In this study, we employed ultrasonography to assess geniohyoid movement before and after rehabilitation in stroke patients with dysphagia, aiming to explore the potential of ultrasound in dysphagia evaluation and rehabilitation.

## Materials and methods

### Study participants

This study was conducted in accordance with the ethical guidelines of the institutional and national research committees, as well as the principles of the 1964 Declaration of Helsinki and its subsequent amendments. Written informed consent was obtained from all participants prior to their inclusion in the study.

*A priori* sample size calculation was performed based on a pilot study (effect size d = 0.8, *α* = 0.05, power = 0.80), resulting in 30 participants per group. A total of 30 stroke patients with dysphagia who underwent rehabilitation treatment between January and March 2024 were enrolled. The patient group comprised 16 males and 14 females, aged 49–78 years (mean age: 66.23 ± 7.89 years). Among them, 18 had ischemic stroke and 12 had hemorrhagic stroke. Mean time since stroke was 4.2 ± 1.8 months. Lesion location was confirmed by MRI or CT in all patients; the majority (22/30, 73.3%) had supratentorial lesions (cortical or subcortical), while the remaining 8 patients (26.7%) had infratentorial (brainstem or cerebellar) strokes. Dysphagia severity was assessed using the Penetration-Aspiration Scale (PAS) from VFSS, with a mean PAS score of 5.3 ± 1.2.

Additionally, 30 age- and sex-matched healthy controls without dysphagia were included (15 males and 15 females, aged 47–75 years; mean age: 65.39 ± 7.68 years). Healthy controls had no history of dysphagia, neurological disorders, or cervical surgery, and were recruited from community health screening.

None of the enrolled patients had a history of weak or absent lip closure, as assessed by clinical oral motor examination and the modified water swallowing test. This finding ensured that any observed swallowing impairments were primarily attributable to pharyngeal phase dysfunction rather than oral containment issues.

No participants were excluded due to the 5 mL water swallow requirement; this volume was chosen to ensure M-mode measurability and is consistent with prior studies (9).

### Inclusion criteria

Patients were included if they met all of the following criteria:

1. Diagnosis of cerebral infarction or cerebral hemorrhage confirmed by MRI or CT;
2. Presence of dysphagia verified by videofluoroscopic swallowing study (VFSS);
3. Ability to swallow 5 mL of water as required for examination, despite having dysphagia confirmed by the modified water swallowing test;
4. Clear consciousness, stable vital signs, and no history of mental illness.

### Exclusion criteria

Patients were excluded based on any of the following:

1. Inability to maintain a seated position with normal head posture;
2. History of any other condition known to affect swallowing function;
3. Impaired consciousness or unstable vital signs precluding completion of swallowing function assessment.

### Equipment

Ultrasonographic examinations were conducted using a high-resolution ultrasound system (LOGIQ E9, GE Healthcare, USA) equipped with a 3.5–5 MHz curvilinear transducer.

### Examination protocol

Subjects were positioned in an upright seated posture with slight cervical extension (approximately 15°–30°). A curvilinear ultrasound transducer was placed midline in the submental region to obtain optimal visualization of the geniohyoid muscle, tongue musculature, mandible, and bilateral hyoid bones in grayscale imaging. Following appropriate depth adjustment and optimal plane selection, the imaging mode was switched to B/M-mode, with the sampling line positioned at the mid-portion of the geniohyoid muscle. Participants were then instructed to perform a single 5 mL water bolus swallow while maintaining position, and the geniohyoid movement trajectory was captured in M-mode during deglutition.

The geniohyoid muscle is the primary contributor to hyoid anterior excursion during swallowing (5), making its displacement a valid kinematic indicator. Key measurements included:

1. Maximum displacement amplitude (mm)
2. Movement duration (s)
3. Average contraction velocity (mm/s), calculated as displacement divided by duration

Ultrasonographic acquisitions and analyses were performed by two experienced ultrasonographer who underwent a standardized training protocol consisting of supervised swallows before the study. Each participant performed three independent 5 mL water swallows, with M-mode measurements repeated three times per swallow, yielding nine measurements that were averaged for analysis. A practice swallow was performed before data collection to reduce variability. Intra-rater reliability (ICC = 0.94, 95% CI: 0.89–0.97) and inter-rater reliability (ICC = 0.91, 95% CI: 0.85–0.95) were assessed using 10 randomly selected scans, indicating excellent agreement. Identical protocols were executed for both pre- and post-treatment assessments in the patient cohort, as well as for control group evaluations.

### Treatment protocol

Neuromuscular electrical stimulation (NMES) therapy was administered using a standardized protocol (10–12). NMES was delivered using a VitalStim® Plus device (Chattanooga Group, USA) with self-adhesive round electrodes (5 cm diameter). Electrodes were placed transcutaneously over the anterior neck, 2 cm lateral to the midline, centered at the level of the hyoid bone, targeting the suprahyoid muscle group. Stimulation parameters were individually adjusted based on patient tolerance and consisted of a biphasic symmetric waveform, frequency of 80 Hz, pulse width of 300 *μ*s, duty cycle of 5 s on and 5 s off, and intensity ranging from 5 to 15 mA (adjusted to produce visible mild muscle contraction without pain). NMES was applied simultaneously with active dry swallowing attempts (functional electrical stimulation).

The treatment schedule was as follows: 30-min sessions, twice daily, 5 sessions per week, for a total intervention period of 6 weeks. All sessions were delivered by the same trained rehabilitation therapist, who was blinded to ultrasound assessments. Adverse events (e.g., skin redness, discomfort) were recorded daily; no serious events occurred.

### Statistical analysis

Statistical analyses were performed using SPSS 25.0. Continuous variables are presented as mean ± standard deviation (SD). Categorical data (gender distribution) were analyzed using Pearson's *χ*² test, while continuous variables (age) were compared using independent samples *t*-tests. For between-group comparisons (case vs. control) and within-group comparisons (pre- vs. post-treatment in the case group), independent samples *t*-tests and paired samples *t*-tests were employed, respectively. All statistical tests were two-tailed, with *P* < 0.05 considered statistically significant.

Given multiple comparisons (three primary outcome parameters across three comparisons), we applied a Bonferroni correction to control for Type I error. The adjusted significance threshold was set at *α* = 0.05/3 = 0.0167. All statistically significant results reported (*P* < 0.05 and *P* < 0.01) remained significant after Bonferroni correction, as the smallest *P* value was < 0.01 (i.e., < 0.0167).

## Results

Demographic characteristics were comparable between the case and control groups, with no statistically significant differences observed in either age distribution or gender composition (*P* > 0.05). (Table 1)

**Table 1:** Comparison of clinical data between case group and control group.

Group	Number	Gender		Age
		Male	Female	
Case group	30	16	14	66.23 ± 7.89
Control group	30	15	15	65.39 ± 7.68
*P* value		＞0.05		＞0.05

### Pre-treatment comparisons

Quantitative analysis revealed significant impairments in geniohyoid muscle kinematics among stroke patients compared to healthy controls prior to intervention. Specifically, the case group demonstrated significantly reduced movement distance (6.88 ± 0.68 mm vs. 8.07 ± 0.76 mm; *P* < 0.05), markedly lower average movement velocity (2.73 ± 0.52 mm/s vs. 8.16 ± 0.72 mm/s; *P* < 0.01), and substantially prolonged movement duration (2.24 ± 0.27 s vs. 0.98 ± 0.15 s; *P* < 0.01).

### Post-treatment outcomes (Pre- vs. Post-treatment)

Following the rehabilitation intervention, the case group exhibited significant improvements in swallowing kinematics. Movement distance increased from 6.88 ± 0.68 mm to 7.56 ± 0.61 mm, representing a mean increase of 9.88% {calculated as [(post-pre)/pre] × 100 using group means; *P* < 0.05}. Movement velocity increased from 2.73 ± 0.52 mm/s to 5.75 ± 0.36 mm/s, a 110.62  improvement (*P* < 0.05). Movement duration decreased from 2.24 ± 0.27 s to 1.31 ± 0.22 s, a 41.52% reduction (*P* < 0.05).

In addition, the Dysphagia Outcome and Severity Scale (DOSS) improved from 3.2 ± 0.6 to 5.1 ± 0.7 (*P* < 0.01), indicating clinically meaningful functional recovery.

### Post-treatment vs. Control Comparisons

**Figure 1:**
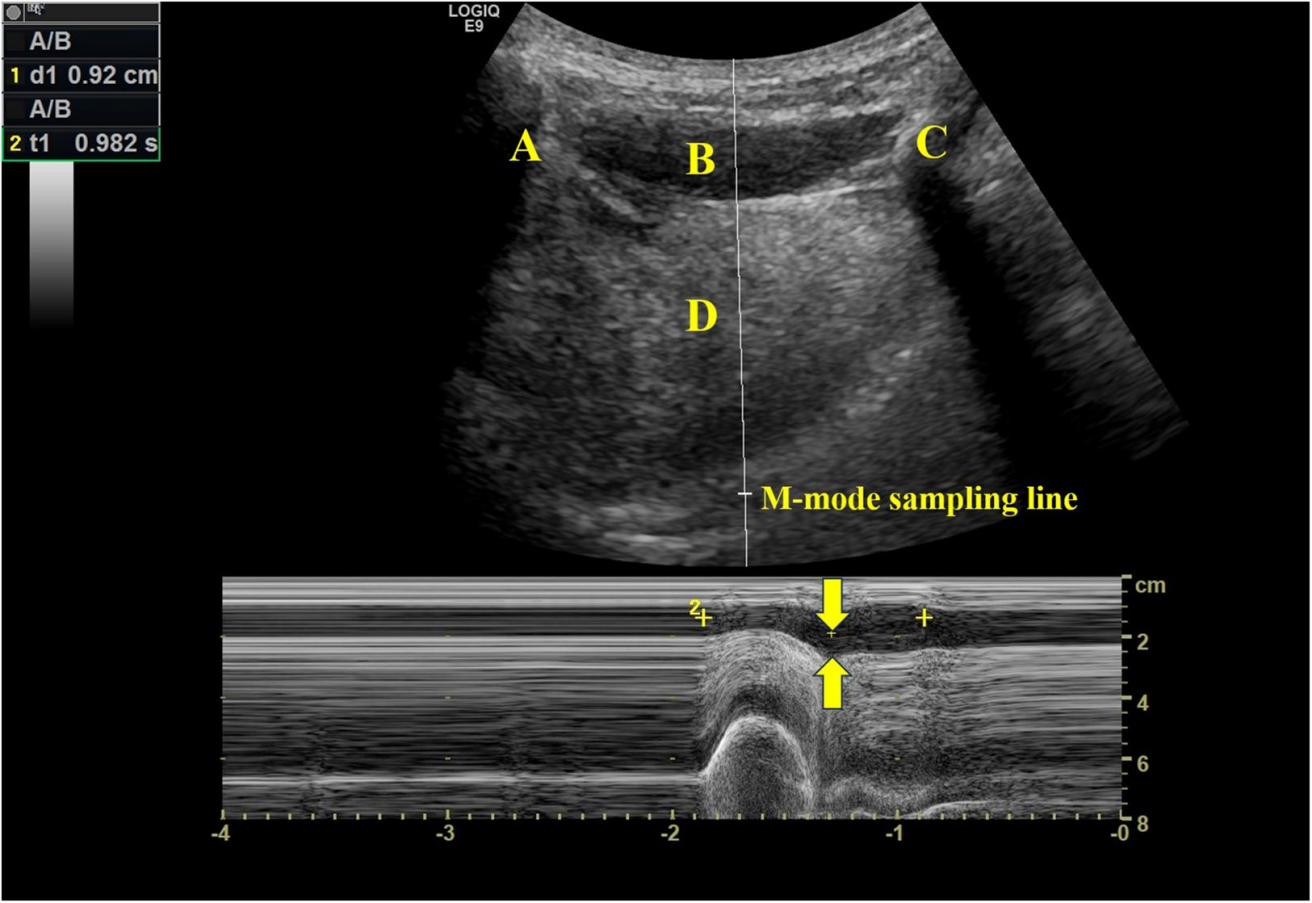
Ultrasound image with four labeled regions A, B, C, and D, and a vertical M-mode sampling line. The lower panel displays an M-mode tracing with yellow arrows indicating measured distances and motion patterns.

**Figure 2:**
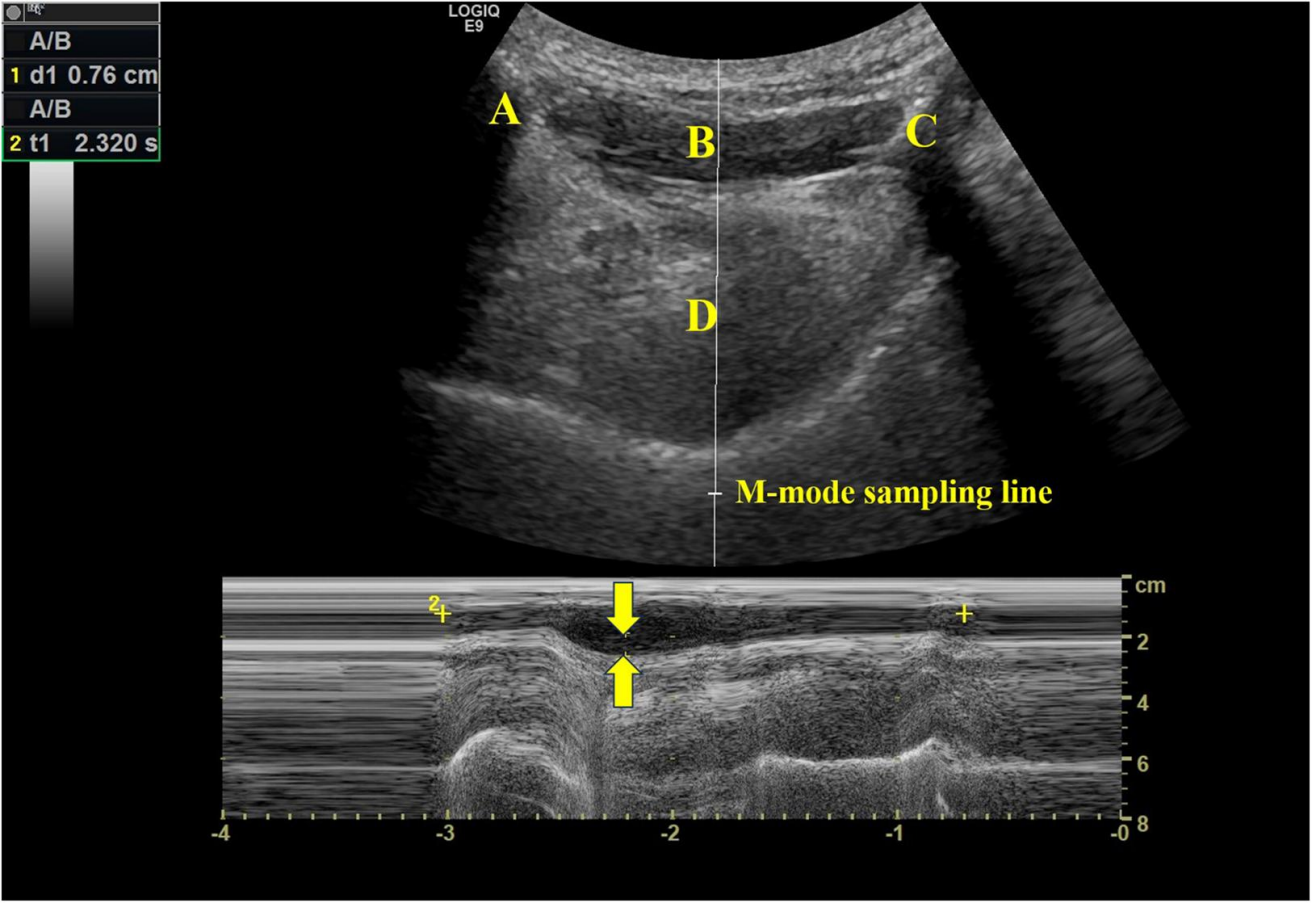
Ultrasound image with labeled anatomical areas A, B, C, and D, and an M-mode sampling line intersecting the regions. The lower section displays a corresponding M-mode tracing, with yellow arrows indicating measurement points. Measurement data include a distance of 0.76 cm and a time of 2.32 s.

**Figure 3:**
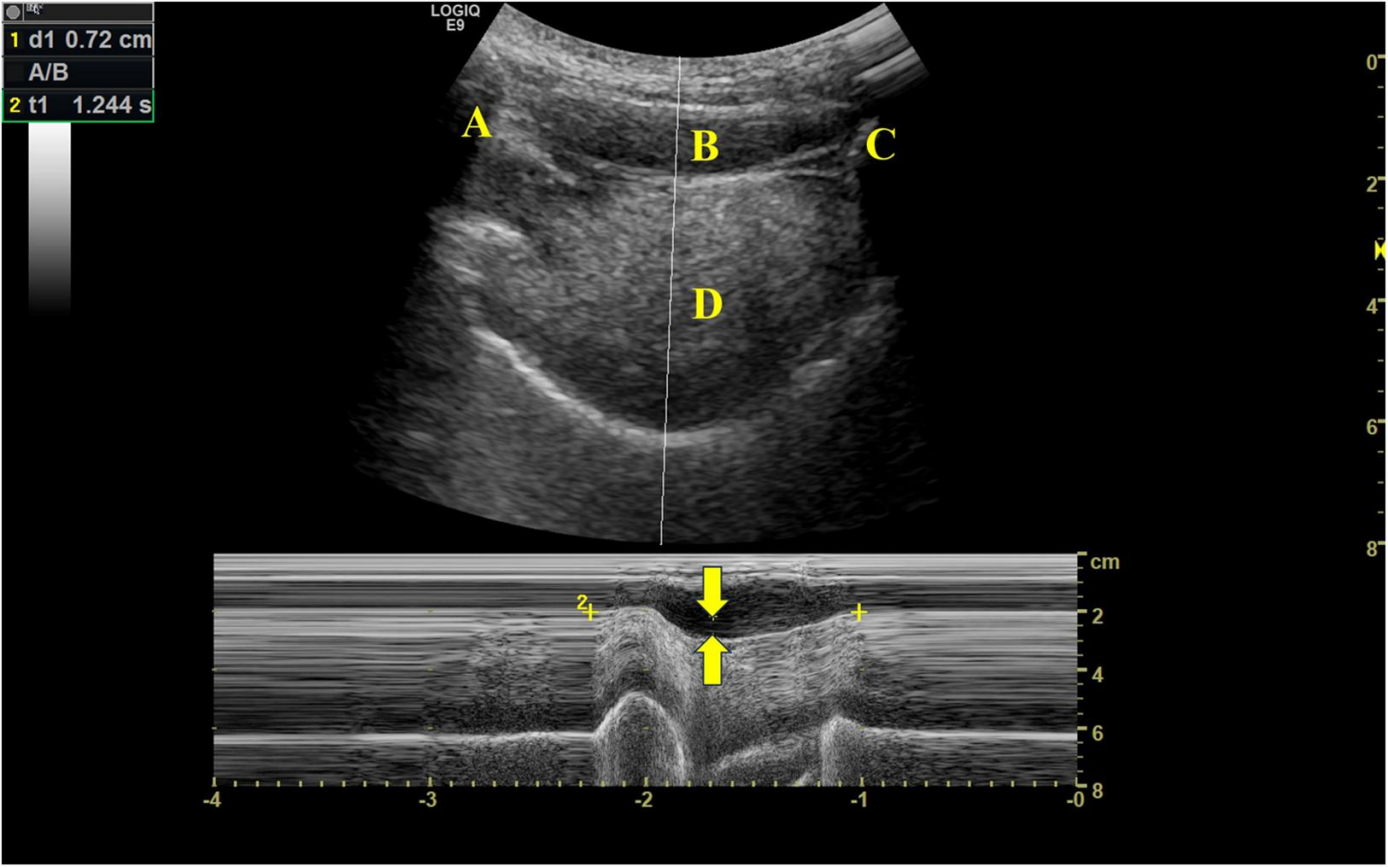
Ultrasound scan showing anatomic structures labeled A, B, C, and D in yellow, with a time-motion (M-mode) graph below. Two bold yellow arrows on the graph indicate measurement points. Top left displays measurements of distance and time.

While rehabilitation produced measurable improvements, residual deficits persisted when compared to healthy controls. Movement distance achieved parity with controls (7.56 ± 0.61 mm vs. 8.07 ± 0.76 mm; *P* > 0.05). However, movement velocity remained significantly slower than controls (5.75 ± 0.36 mm/s vs. 8.16 ± 0.72 mm/s; *P* < 0.05), and movement duration continued to exceed control values (1.31 ± 0.22 s vs. 0.98 ± 0.15 s; *P* < 0.05). (Table 2) (Figures 1–3).

**Table 2:** Comparison of geniohyoid movement parameters—case group (before and after treatment) vs. control group.

Group	Movement distance (mm)	Movement time (s)	Average Movement speed (mm/s)
Case group (Before treatment)	6.88 ± 0.68	2.24 ± 0.27	2.73 ± 0.52
Case group (After treatment)	7.56 ± 0.61	1.31 ± 0.22	5.75 ± 0.36
Control group	8.07 ± 0.76	0.98 ± 0.15	8.16 ± 0.72
*P* values			
Case vs. Control (Before)	<0.05	<0.01	<0.01
Before vs. After (Case)	<0.05	<0.05	<0.05
Case vs. Control (After)	>0.05	<0.05	<0.05

Data are presented as mean ± standard deviation. *P* values indicate statistical significance between the specified group comparisons. “Before” and “After” refer to pre- and post-treatment measurements within the case group. The control group measurements are consistent across both time points (no treatment applied).

## Discussion

The primary aim of this study was to determine whether ultrasonography can quantitatively detect changes in geniohyoid movement parameters in stroke patients with dysphagia following rehabilitation. Our results demonstrate that ultrasound-derived measures of displacement, velocity, and duration all improved significantly after six weeks of NMES. Specifically, mean movement distance increased from 6.88 mm to 7.56 mm (a 9.88% increase, *P* < 0.05), mean velocity rose from 2.73 mm/s to 5.75 mm/s (a 110.62% increase, *P* < 0.05), and mean duration decreased from 2.24 s to 1.31 s (a 41.52% reduction, *P* < 0.05). Notably, post-treatment displacement (7.56 mm) was not significantly different from healthy controls (8.07 mm, *P* > 0.05), indicating a return to normal range. Thus, ultrasonography is sensitive to rehabilitation-induced changes and can serve as a practical tool for monitoring swallowing kinematics.

Consistent with previous reports (13–15), our pre-treatment comparisons confirmed that stroke patients exhibit reduced geniohyoid displacement (6.88 vs. 8.07 mm), slower velocity (2.73 vs. 8.16 mm/s), and prolonged duration (2.24 vs. 0.98 s) relative to healthy controls. These abnormalities likely reflect stroke-induced neurological impairment affecting the swallowing musculature. The geniohyoid muscle, a key suprahyoid muscle, plays a pivotal role in hyoid anterior excursion, UES opening, and epiglottic inversion (5). Its dysfunction directly contributes to clinically significant dysphagia (16).

Following NMES intervention, we observed significant improvements in all three kinematic parameters. Our NMES protocol (VitalStim® Plus, 80 Hz, 300 μs, 5–15 mA, combined with active swallowing) is consistent with recent guidelines that advocate for task-specific electrical stimulation to enhance neuroplasticity (10–12). The observed improvements are in line with other studies showing that NMES can increase hyoid displacement and reduce swallowing duration in post-stroke dysphagia (1, 9, 17, 18).

A novel finding of our study is the differential recovery pattern: displacement normalized to control levels (7.56 vs. 8.07 mm, *P* > 0.05), while movement duration (1.31 vs. 0.98 s, *P* < 0.05) and velocity (5.75 vs. 8.16 mm/s, *P* < 0.05) remained significantly impaired. This hierarchy-displacement recovering faster than temporal parameters-suggests that the neural circuits controlling movement amplitude may be more amenable to early rehabilitation than those controlling timing and coordination. To our knowledge, this is the first documentation of such temporally staggered restitution in post-stroke deglutition. Recent studies have also highlighted the importance of movement timing in swallowing safety (19, 20).

This observation has clinical implications. Rehabilitation efforts might initially focus on restoring movement amplitude, but additional or prolonged training may be required to improve movement speed and coordination. Clinicians using ultrasound can monitor not only displacement but also duration, which may serve as a more sensitive indicator of residual impairment. Our future research will involve a larger longitudinal study with weekly ultrasound assessments to track the trajectory of each kinematic parameter, correlate them with VFSS-derived aspiration measures, and develop a parameter-specific NMES protocol (e.g., adjusting stimulation frequency or electrode placement when duration remains prolonged).

The persistent prolongation of movement duration (1.31 s vs. 0.98 s) despite normalized displacement accounts for the residual velocity deficit. This temporal parameter appears particularly sensitive to residual neuromuscular impairment, though it is not necessarily the single “best” marker. Nonetheless, prolonged duration may correlate with delayed airway protection, a hypothesis that warrants further investigation using simultaneous VFSS-ultrasound recordings (21).

## Caveats and future directions

Although NMES may have contributed to the observed improvements, spontaneous neurological recovery cannot be excluded. The observed changes likely reflect a combination of NMES and natural neuroplasticity. Future studies should integrate ultrasound with VFSS or FEES to directly link kinematic parameters with aspiration risk. Additionally, emerging evidence suggests that ultrasound can also be used to assess muscle thickness and echo intensity, which may provide complementary information on muscle quality (7, 8, 22).

## Limitations and conclusions

This study has limitations, including a modest sample size and a relatively short observation period, which precluded longitudinal assessment through complete functional recovery. Nevertheless, ultrasonography provides a reliable quantitative method for assessing geniohyoid movement kinematics in stroke-related dysphagia, offering objective parameters to guide clinical rehabilitation. Future investigations with larger cohorts and extended follow-up durations are needed to validate these preliminary findings and further elucidate the long-term recovery trajectory.

## Data Availability

The raw data supporting the conclusions of this article will be made available by the authors, without undue reservation.
